# PD-L1 immunohistochemical expression considering HPV status in oropharyngeal squamous cell carcinoma

**DOI:** 10.1590/1807-3107bor-2024.vol38.0095

**Published:** 2024-09-30

**Authors:** Raíssa Soares dos ANJOS, Marianne de Vasconcelos CARVALHO, Rayanna Thayse Florêncio COSTA, Belmiro Cavalcanti do Egito VASCONCELOS, Sandra Lúcia Dantas MORAES, Eduardo Piza PELLIZZER

**Affiliations:** (a) Universidade de Pernambuco – UPE, School of Dentistry, Department of Oral and Maxillofacial Pathology, Recife, PE, Brazil.; (b) Universidade de Pernambuco – UPE, School of Dentistry, Department of Prosthodontics, Recife, PE, Brazil.; (c) Universidade de Pernambuco – UPE, School of Dentistry, Department of Oral and Maxillofacial Surgery, Recife, PE, Brazil.; (d) Universidade Estadual Paulista – Unesp, Dental School of Araçatuba, Department of Dental Materials and Prosthodontics, Araçatuba, SP, Brazil.

**Keywords:** Human Papillomavirus Viruses, Squamous Cell Carcinoma of Head and Neck, Tumor Microenvironment

## Abstract

This systematic review aims to determine whether the presence of human papillomavirus (HPV) influences the immunohistochemical expression of programmed cell death-1 ligand (PD-L1) in oropharyngeal squamous cell carcinoma (OPSCC). PD-L1 immunohistochemical expression varies in OPSCC, and the presence of HPV is a plausible explanation for this variability. Comprehending these findings is crucial, as high PD-L1 expression in the tumor microenvironment of OPSCC can help identify patient subgroups that could be suitable for immunotherapy. Therefore, a systematic review was conducted following PRISMA guidelines (CRD42023437800). An electronic literature search was performed without time or language restrictions. The search included PubMed/MEDLINE, Embase, Scopus, Web of Science, https://clinictrials.gov, and relevant journals. A meta-analysis was performed using RStudio. Fourteen studies involving 1,629 participants were included. The sample consisted predominantly of males (81.26%) with a mean age of 58.3 years. Concerning clinical and pathological characteristics, the most frequently described anatomical location was the tonsils (68.54%), and most participants were either current or former smokers (78%) and alcohol users (79%). Advanced TNM IV was the most common stage. Regarding histopathological characteristics, HPV 16 was the only type mentioned, and half of the cases were detected through immunohistochemistry. The SP142 clone (35.7%) and the pattern of membrane immunostaining in tumor cells (71%) were the most commonly employed methods. The most prevalent findings were positive expression of PD-L1 (64.28%) and negative HPV status (57.14%). The association between PD-L1 positivity and HPV positivity (78.57%) was confirmed by meta-analysis. The conclusion was that HPV-positive status has an impact on immunohistochemical expression of PD-L1 in OPSCC.

## Introduction

Oropharyngeal cancer represents a significant public health concern. In 2020, the International Agency for Research on Cancer (IARC) reported approximately 19.3 million new cases of malignant neoplasms worldwide each year. Among these, 98,421 (0.5%) cases were diagnosed as oropharyngeal lesions,^1^ with over 90% of them histologically classified as oropharyngeal squamous cell carcinoma (OPSCC).^2^

Human papillomavirus (HPV) infection represents one of the primary etiologic factors for OPSCC.^3^ More than 90% of OPSCC cases are associated with high-risk HPV type 16.^4^ Furthermore, the presence of HPV can significantly impact the prognosis and oncological treatment outcomes.^3^ The prognosis is better for patients with HPV-positive OPSCC (HPV+OPSCC) than for those with HPV-negative OPSCC (HPV-OPSCC).^5^

Additionally, limited analyses suggest variations in the response to immunotherapy between patients with HPV+OPSCC and HPV-OPSCC.^6,7^ This disparity may be attributed to the characteristics of the tumor microenvironment (TME) and to the actions of immunoregulatory agents, including programmed cell death receptor-1 (PD-1) and its ligand (PD-L1).^8^

Understanding PD-L1 immunohistochemical expression within the TME in OPSCC can aid in the identification of patient subgroups suitable for immunotherapy.^9,10^ In OPSCC, the immunohistochemical expression of the PD-1/PD-L1 axis is heterogeneous, and the presence of HPV is one plausible explanation for that.^11^ Nevertheless, the idea that the presence of HPV in OPSCC influences PD-L1 immunohistochemical expression remains controversial among various studies.

The objective of this systematic review is to ascertain whether there is an association between HPV status and PD-L1 immunohistochemical expression in OPSCC.

## Methods

### Data sources and search strategies

This systematic review was registered in the International Prospective Register of Systematic Reviews (CRD42023437800) and followed the Preferred Reporting Items for Systematic Reviews and Meta-analyses (PRISMA) guidelines.^12^

For inclusion of studies in this systematic review, the population, exposure, comparison, and outcome (PECO) strategy was employed: (P) patients diagnosed with OPSCC; (E) PD-L1 immunohistochemical expression; (C) absence of PD-L1 immunohistochemical expression; and (O) the risk rate of HPV expression. The research question posed was, “Does the presence of HPV in OPSCC impact PD-L1 immunohistochemical expression?”.

### Eligibility criteria and study selection

The inclusion criteria for studies in this systematic review were as follows: a) clinical studies (randomized and non-randomized clinical trials, retrospective studies, cohort studies, and longitudinal and cross-sectional studies); b) participants with a histologic diagnosis of OPSCC; c) assessment of PD-L1 and HPV status; d) studies reporting the association between PD-L1 status and HPV status in participants; e) description of PD-L1 immunohistochemical expression based on the combined positivity score (CPS) and tumor proportion score (TPS). Additionally, no distinctions were made based on sex, age, race/skin color, publication period, or language. Participants with a history of prior immunotherapy were excluded from the study. In those cases in which multiple studies included the same group of participants, preference was given to those with more recent data or those meeting the inclusion criteria.

The search was conducted in pairs in the PubMed/MEDLINE, Scopus, Web of Science, and Embase databases. Additionally, non-peer-reviewed literature was accessed at https://clinicaltrials.gov. The reference lists of included articles were scrutinized as a source, and manual searches for articles were carried out in the journals *Frontiers in Oncology*, *Oral Oncology,* and *Oncotarget* (Table 1).

**Table 1:** Search strategy for each database and journal.

Databases and journals	Search strategy	Filter
Database		
PubMed/MEDLINE	(HPV OR human papillomavirus) AND (PD-L1 OR PDL1 OR checkpoint OR immunoexpression OR programmed cell death ligand 1 OR tumor microenvironment) AND (oropharyngeal cancer OR oropharyngeal carcinoma OR oropharyngeal neoplasm OR oropharyngeal tumor OR oropharyngeal squamous cell carcinoma OR OPSCC)	No filters
Scopus	(HPV OR ‘human papillomavirus’) AND (PD-L1 OR PDL1 OR checkpoint OR immunoexpression OR ‘programmed cell death ligand 1’ OR ‘tumor microenvironment’) AND (‘oropharyngeal cancer’ OR ‘oropharyngeal carcinoma’ OR ‘oropharyngeal neoplasm’ OR ‘oropharyngeal tumor’ OR ‘oropharyngeal squamous cell carcinoma’ OR OPSCC)	All Fields
Web of Science	ALL=((HPV OR human papillomavirus) AND (PD-L1 OR PDL1 OR checkpoint OR immunoexpression OR programmed cell death ligand 1 OR tumor microenvironment) AND (oropharyngeal cancer OR oropharyngeal carcinoma OR oropharyngeal neoplasm OR oropharyngeal tumor OR oropharyngeal squamous cell carcinoma OR OPSCC))	All Fields
Embase	(‘hpv’/exp OR hpv OR ‘human papillomavirus’ OR ((‘human’/exp OR human) AND papillomavirus)) AND (‘pd l1’ OR pdl1 OR ‘checkpoint’/exp OR checkpoint OR ‘immunoexpression’/exp OR immunoexpression OR ‘programmed cell death ligand 1’/exp OR ‘programmed cell death ligand 1’ OR (programmed AND (‘cell’/exp OR cell) AND (‘death’/exp OR death) AND (‘ligand’/exp OR ligand) AND (‘1’/exp OR 1)) OR ‘tumor microenvironment’/exp OR ‘tumor microenvironment’ OR ((‘tumor’/exp OR tumor) AND (‘microenvironment’/exp OR microenvironment))) AND (‘oropharyngeal cancer’/exp OR ‘oropharyngeal cancer’ OR (oropharyngeal AND (‘cancer’/exp OR cancer)) OR ‘oropharyngeal carcinoma’/exp OR ‘oropharyngeal carcinoma’ OR (oropharyngeal AND (‘carcinoma’/exp OR carcinoma)) OR ‘oropharyngeal neoplasm’ OR (oropharyngeal AND (‘neoplasm’/exp OR neoplasm)) OR ‘oropharyngeal tumor’/exp OR ‘oropharyngeal tumor’ OR (oropharyngeal AND (‘tumor’/exp OR tumor)) OR ‘oropharyngeal squamous cell carcinoma’/exp OR ‘oropharyngeal squamous cell carcinoma’ OR (oropharyngeal AND squamous AND (‘cell’/exp OR cell) AND (‘carcinoma’/exp OR carcinoma)) OR opscc)	All Fields
https://clinicaltrials.gov	Condition or disease: oropharyngeal cancer OR oropharyngeal carcinoma OR oropharyngeal neoplasm OR oropharyngeal tumor OR oropharyngeal squamous cell carcinoma OR OPSCC	No
	Other terms: HPV OR human papillomavirus	Fields
	Intervention/Treatment: PD-L1 OR PDL1 OR checkpoint OR immunoexpression OR programmed cell death ligand 1 OR tumor microenvironment	
Journal		
Frontiers in Oncology	PD-L1 AND OPSCC AND HPV	No Filters
Oral Oncology	PD-L1 AND OPSCC AND HPV	No Fields
Oncotarget	PD-L1 AND OPSCC AND HPV	No Fields

The search strategy applied in each database is detailed in Table 1. After searching each database, duplicate articles were removed using reference management software (Reference Manager; Mendeley Ltd, Elsevier Inc), and the initial study selection was based on title and abstract. Subsequently, a comprehensive review of the full articles was conducted.

### Data extraction and analysis

**Figure 1:**
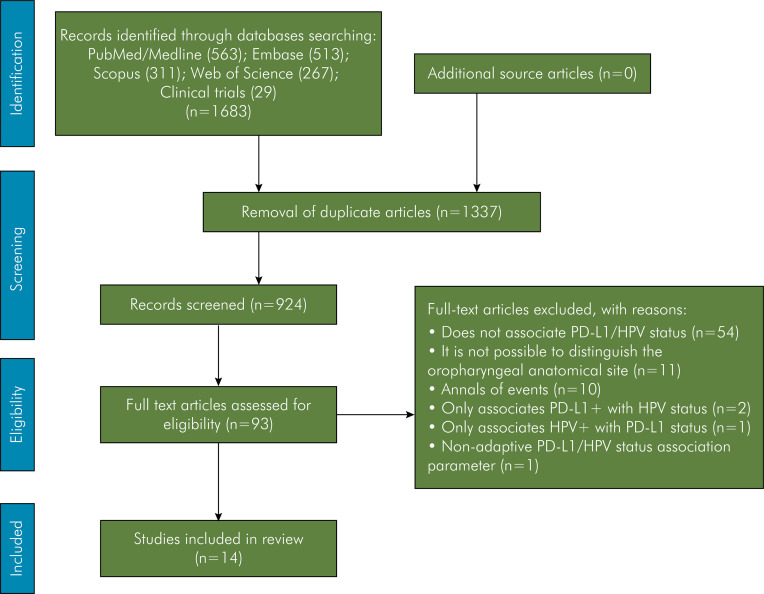
Flow diagram describing the studies selection.

The inclusion of studies and extraction were manually performed by one researcher (R.S.A.) and reviewed by a second researcher (R.T.F.C.). The data collected by these two researchers were analyzed by a third researcher (M.V.C.), and consensus was achieved through discussion. The article selection process is presented in Figure 1.

The variables extracted from the studies included author, year of publication, country, study design, number of participants, sex, age, clinical and pathologic characteristics (anatomical location, smoking, alcohol consumption, TNM classification of malignant tumors (TNM), American Joint Committee on Cancer (AJCC) TNM system (seventh and eighth editions), histologic classification, HPV subtype, HPV assessment technique, PD-L1 antibody (clone) and concentration, PD-L1 immunohistochemistry (IHC) technique, PD-L1 IHC cell labeling pattern, description of PD-L1 immunohistochemical expression (CPS and TPS), PD-L1 status (positive and negative), HPV status (positive and negative), and the association between PD-L1 and HPV status.

### Risk of bias

The risk of bias in each study was assessed using the JBI Critical Appraisal Checklist for Analytical Cohort Studies and conducted by two researchers (R.S.A. and R.T.F.C.). This instrument evaluates the methodological quality of a study and determines the extent to which it addresses the potential of bias in its design, conduct, and analysis.^12^

### Meta-analysis

Meta-analysis was performed utilizing RStudio and Excel 365 software, and computations were carried out with the meta package using the Mantel-Haenszel method. The experimental group consisted of HPV+ and PD-L1+, while the control group included HPV- and PD-L1+ individuals. Two authors evaluated the SP142 and 22C3 clones,^13,14^ but for the meta-analysis, only the values referring to the SP142 clone were considered. Other authors evaluated immunohistochemical expression patterns in tumor and immune cells,^15,16^ but for the meta-analysis, only values related to the immunohistochemical expression pattern in tumor cells were considered.

## Results

### Study selection

The electronic search, as described in the methodology, was conducted until September 2023, yielding a total of 1,683 articles (PubMed/MEDLINE, 563; Embase, 513; Scopus, 311; Web of Science, 267), with an additional 29 records obtained from https://clinictrials.gov. After removing duplicates, 1,337 articles remained. Subsequently, a preliminary screening of titles and abstracts was performed, and inclusion criteria were applied, resulting in 93 articles eligible for full-text examination. After a thorough review, 79 articles were excluded for various reasons, including failure to establish an association between PD-L1 and HPV status (n = 54), inability to specify the anatomical site as the oropharynx (n = 11), conference proceedings (n = 10), sole association of PD-L1 positive (PD-L1+) with HPV status (n = 2), sole association of HPV positive (HPV+) with PD-L1 status (n=1), and the utilization of unadaptable parameters for assessing the association of PD-L1/HPV status (n = 1). Thus, a total of 14 articles^11,13^ met the inclusion criteria in this review.

### Study characteristics

The characteristics and outcomes of the 14 selected studies are comprehensively outlined in Table 2. These studies were categorized as retrospective,^13^ cohort,^11,20,23,24^ and cross-sectional.^25^ In total, the evaluation involved 1,629 participants, and 81.26% of them were male.^11,13^ The age of the participants ranged from 34 to 89 years, with a mean of 58.3 years.^11,16,17,20,21,23^

**Table 2:** General data of the selected studies and clinicopathologic characteristics of the sample.

Author and year of publication	Country	Study design	Sample size	Sex (%)	Mean age (range)	Anatomical location (%)	Smoking (%)	Drinking (%)	T Staging	N Staging	M Staging
Kim et al, 2016^17^	South Korea	Retrospective	133	M – 120 (90.2)	57.5	Tonsil – 104 (78.19)	Never – 51	-	T1 – 33	N0 – 22	-
				F – 13	(35-80)	Soft palate – 4 (3)	-38		T2 – 74	N1 – 35	
				(9.8)		Base of the tongue – 9 (6.76)	Smoker – 81 (62)		T3 – 15	N2 – 68	
						Other sites – 16			T4 – 11	N3 – 8	
						(12.03)					
Hong et al, 2016^23^	Australia	Cohort	99	M – 79	58	Tonsil – 99 (100)	Never – 27	Never – 12	T1 – 16	N0 – 33	-
				(79.79)	(34-83)		(27.27)	(12.12)	T2 – 40	N1 – 23	
				F – 20			Ex-smoker – 30 (30.30)	Ex- alcoholic – 7	T3 – 28	N2 – 37	
				(20.21)			Smoker – 42 (42.42)	(7.07)	T4 – 15	N3 – 6	
								alcoholic – 77			
								(77.77)			
Meulenaere et al, 2017^14^	Belgium	Retrospective	99	M – 82	-	Tonsil – 43	-	-	T1 and T2 – 56	N0 – 30	-
				(82.82)		(43.43)			T3 and T4 – 43	N1 – 14	
				F – 17		Base of the tongue – 12 (12.12)				N2 and 3 – 55	
				(17.18)		Other sites – 23 (23.23)					
						Multiple sites – 21 (21.21)					
			
Fukushima et al, 2018^15^	Japan	Retrospective	92	M – 77	-	Tonsil – 67 (72.82)	Never – 20	Never – 21 (22.82)	T1 and T2 – 57	N0 and 1 – 44	-
				(83.69)	(36-89)	Base of the tongue – 12 (13.04)	(21.73)	Ex-alcoholic / Alcoholic –	T3 and T4 – 35	N2 and 3 – 48	
				F – 15		Other sites – 13 (14.13)	Ex-smoker/Smoker – 72 (78.26)	71 (77.17)			
				(16.31)							
Kwon et al, 2018^24^	South Korea	Cohort	79	M – 68	-	Tonsil – 79 (100)	<20 – 23 (29.11)	<14 – 32 (40.5)	T1 and T2 – 47	N0 – 18	-
				-86			≥20 – 56 (70.88)	≥14 – 47 (59.5)	T3 and T4 – 32	N1 and 2 – 61	
				F – 11							
				-14							
Hong et al, 2019^20^	Australia	Cohort	214	M – 166	59	-	Never – 23 (10.74)	-	T1 – 38	N0 – 90	-
				(77.57)	(31-83)		Ex-smoker – 64 (29.9)		T2 – 74	N1 – 41	
				F – 48			Smoker – 127		T3 – 69	N2 – 69	
				(22.43)			(59.34)		T4 – 33	N3 – 13	
Jeong et al, 2020^13^	South Korea	Retrospective	109	M – 97	-	Tonsil – 62 (56.88)	Never – 27 (24.77)	-	T1 and T2 – 78	N0 – 17	-
				-89	(39 a 67)	Other sites – 44 (40.36)	Smoker – 79 (72.47)		T3 and T4 – 28	N1,2 and 3 – 89	
				F – 9							
				-11							
Lilja-Fischer et al, 2020^11^	Denmark	Cohort	303	M – 217	59	Tonsil – 205	Never – 48 (15.84)	-	T1 and T2 – 182	N0 – 81	M0 – 303
				(71.61)	(53-65)	(67.65)	Ex-smoker – 86		T3 and T4 – 121	N1 – 59	
				F – 86		Base of the tongue – 68 (22.44)	(28.38)			N2 and 3 – 163	
				(28.39)		Other sites – 30 (9.9)	Smoker – 168				
							(55.44)				
							NA – 1				
							(0.33)				
Gurin et al, 2020^16^	Czech Republic	Retrospective	65	M – 53	59	-	-	-	T1 – 0	N0 and 1 – 11	-
				(81.53)	(54-63)				T2 – 9	N2 – 46	
				F – 12					T3 – 13	N3 – 8	
				(18.47)					T4 – 43		
Atipas et al, 2023^22^	Thailand	Retrospective	160	-	-	-	-	-	-	-	-
Schmidl et al, 2023^18^	Germany	Retrospective	47	M – 37	-	-	-	-	T1 and T2 – 39	N0 – 19	M0 – 47
				(78.72)					T3 and T4 – 8	N1, 2 and 3 – 28	
				F – 10							
				(21.28)							
Zhu et al, 2023^19^	China	Retrospective	100	-	-	-	-	-	-	-	-
Lee et al, 2023^21^	China	Retrospective	116	M – 103	57.3	Tonsil – 47 (40.51)	Yes – 77 (66.37)	Yes – 84 (72.41)	T1 – 42	N0 – 61	-
				(88.79)	(47.4-67.2)	Soft palate – 35 (30.17)	No – 39 (33.62)	No – 32 (27.58)	T2 – 38	N1 – 6	
				F – 13		Base of the tongue – 27 (23.27)			T3 – 11	N2 – 47	
				(11.21)		Other sites – 6			T4 – 25	N3 – 2	
						(5.17)					
Kareer et al, 2023^25^	India	Cross-sectional	13	-	-	-	-	-	-	-	-

AJCC: American Joint Committee on Cancer; F: Female; M: Male (M). *Kwon et al.^24^ stratified smoking as < or ≥ 20 packs of cigarettes per year and drinking as < or ≥ 14 doses per week.

### Clinical and pathologic characteristics

Regarding clinical and pathologic characteristics, all studies included focused on the oropharynx and nearly half of the studies categorized the anatomical location as the tonsil (68.54%), base of the tongue (12.42%), soft palate (3.78%), other sites (12.81%), and multiple sites (2.03%).^11,13^ The term “Oropharynx, NOS” was used by one author and meant “not otherwise specified.” ^17^The other authors who described the “anatomical location” data used the term “other sites” to refer to regions that were not “tonsil”, “base of the tongue,” and “soft palate.” ^14,17,23^ As the terms have the same meaning, the values for “Oropharynx, NOS” and “other sites” were combined. One author used the term “multiple sites” to denote that two or more anatomical locations (tonsil, base of the tongue, tonsillar pillars, posterior wall, vallecula) would be affected simultaneously. It is possible to infer from the text the prevalence percentage of individuals affected by “multiple sites,” but it is not possible to specify which combinations of locations were found by the author.^14^ Two studies focused exclusively on tonsil cases.^23,24^ Smoking habits were categorized as never/former smoker/smoker,^11,13,15,17,20,23^ < or ≥ 20 packs per year,^24^ and yes or no.^21^ Alcohol consumption was described as never/former alcoholic/alcoholic,^15,23^ < or ≥ 14 drinks per week,^24^ and yes or no.^21^ Most participants were identified as smokers or former smokers (78%) and alcoholics or former alcoholics (79%).

The TNM system was subdivided into tumor (T), lymph nodes (N), and metastasis (M) in all articles that reported these data. However, the M value was only described by two authors.^11,18^ The AJCC system was reported in the seventh^11,14,15,17,20,21,23,24^ and eighth editions,^13,16^ and stage IV was the most common in all studies that provided this information. All authors included described the number and percentage of HPV+ cases.^11,13^ In some articles, data on sex, age, and clinicopathologic characteristics could not be obtained due to the lack of information on the oropharynx.^19,22,25^

### Histopathologic characteristics and analysis of HPV and PD-L1 in OPSCC

Table 3 describes the histopathologic characteristics regarding the classification and tumor characterization of HPV/PD-L1. The most frequently used histologic classification was well/moderately/poorly differentiated.^11,13,14,16,20,23,24^ The reported values for this parameter varied among the studies. Of note, nearly half of the selected articles did not address this specific information.^15,17^

**Table 3:** Histopathologic characteristics regarding tumor classification and characterization of HPV/PD-L1.

Author and year of publication	Histologic classification (%)	HPV subtype	HPV + (%)	HPV assessment technique	PD-L1 antibody (clone and concentration)	PD-L1 IHC technique	PD-L1 IHC Cell labeling pattern	CPS reference values	TPS reference values
Kim et al, 2016^17^	-	p16	89/133 (67)	IHC	Clone 5H1 (1:1000)	FFPE	TCM ≥ 20	-	-
Hong et al, 2016^23^	Well – 7	p16	48/99 (48.5)	PCR	Clone E13LN (1:200)	FFPE	TCM > 1%	-	-
	Moderate – 51								
	Poor – 41								
Meulenaere et al, 2017^14^	Well / Moderate – 67	p16	19/100 (19)	ISH	Clone SP142 (NR)	FFPE	TCM ≥ 5%	-	-
	Poor – 19				Clone 22C3 (NR)				
	Basaloid – 13								
Fukushima et al, 2018^15^	-	p16	45/92 (49)	IHC	Clone SP142	FFPE	TCM + ICM ≥ 5%	-	-
					(1:100)				
Kwon et al, 2018^24^	Well / Moderate – 53	-	28/79 (35)	PANArray HPV chip test	Clone SP142 (1:25)	TMA	TCM ≥ 5%	-	-
	Poor – 26								
Hong et al, 2019^20^	Well – 53	p16	81/214 (38)	PCR + IHC	Clone E1L3N	FFPE	TCM ≥ 1%	-	-
	Moderate / Poor – 161				(1:200)				
Jeong et al, 2020^13^	Well – 5	-	45/109 (41)	PCR	Clone SP142 (NR)	FFPE	TCM ≥50% (SP263) and ≥10% (SP142)	-	-
	Moderate – 90								
	Poor – 11				Clone SP263 (NR)				
Lilja-Fischer et al, 2020^11^	Well / Moderate – 136	p16	156/303 (51.5)	IHC	Clone 22C3 (NR)	FFPE	TCM+ICM	≥1	*
	Poor – 167								
Gurin et al, 2020^16^	Well – 7	p16	45/65 (69)	-	Clone 28-8 (1:200)	FFPE	TCM ≥ 5%	-	-
	Moderate – 24								
	Poor – 8								
	Not informed – 26								
Atipas et al, 2023^22^	-	p16	27/160 (17)	IHC	Clone 22C3 (NR)	TMA	TCM + ICM	≥ 1	-
Schmidl et al, 2023^18^	-	p16	25/47 (53)	IHC + ISH	Clone SP142 (NR)	FFPE	TCM	-	≥ 1%
Zhu et al, 2023^19^	-	p16	50/100 (50)	PCR	NR (1:100)	FFPE	TCM ≥ 5 %	-	-
Lee et al, 2023^21^	Well / Moderate – 86	p16	25/116 (21.5)	IHC	Clone 22C3 (1:50)	TMA	TCM + ICM	≥ 20	-
	Poor – 30								
Kareer et al, 2023^25^	-	p16	8/13 (61.5)	IHC	Clone CAL10 (NR)	-	TCM ≥10%	-	-

Number of PD-L1-positive tumor cells/lymphocytes/macrophages divided by the total number of viable tumor cells (CPS); formalin-fixed paraffin-embedded tumor sections in µm (FFPE); immune cell membrane (ICM); immunohistochemistry (IHC); *in situ* hybridization (ISH); nucleic acid-based peptide test that can detect 32 HPV subtypes simultaneously (PANArray HPV chip test); polymerase chain reaction (PCR); HPV subtype 16 (P16); tissue microarray (TMA); tumor cell membrane (TCM); percentage of viable tumor cells showing partial or complete membrane immunoreactivity of PD-L1 at any intensity (TPS).

*Lilja-Fischer et al.,^11^ a specific value for TPS was not provided; it was simply defined as “percentage of neoplastic cells expressing PD-L1 at any intensity.

All articles that reported on the HPV subtype specified p16.^11,14,15,17^ All authors included described the number and percentage of HPV+ cases.^11,13^ Various techniques were employed to assess HPV, including immunohistochemistry (IHC) (50%),^11,15,17,18,21,22,25^ real-time polymerase chain reaction (PCR) (21.42%),^13,19,23^ and one article utilized both methods (7.14%).^20^ Two articles used in situ hybridization (ISH) (14.28%),^14,18^ but one combined ISH and IHC (7.14%).^18^ The PANArray HPV chip test was utilized in one study (7.14%),^24^ while other article did not specify the method for virus identification (14.28%).^16^

Diverse PD-L1 antibody clones were identified. The SP142 clone was the most frequently employed (35.71%),^13^ followed by the 22C3 clone (28.57%)^11,14,21,22^ and the E13LN clone (14.28%).^20,23^ Additional clones used included 5H1 (7.14%),^17^ 28-8 (7.14%),^16^ SP263 (7.14%),^13^ and CAL10 (7.14%).^25^ One author did not specify the clone employed but described a 1:100 dilution.^19^ Other studies mentioned dilutions of 1:1000,^17^ 1:200,^16,20,23^ 1:100,^15^ 1:50,^21^ and 1:25.^24^ Only two studies utilized the same antibody (E13LN) at an identical dilution (1:200).^20,23^

Immunohistochemical assessment of PD-L1 was predominantly conducted on formalin-fixed paraffin-embedded tumor sections (FFPE) in most of the studies (64.28%).^11,13,15^ Other studies employed tissue microarray (TMA) (21.42%),^21,22,24^ while some did not specify the method used (14.28%).^14,25^

The PD-L1 immunoreactivity pattern was predominantly evaluated based on membrane staining in tumor cells (TCM) (71%)^13,14,16^ and membrane staining in immune cells (ICM) (29%).^11,15,21,22^ The reference values for defining immunoreactivity varied among the studies, with thresholds including ≥1%, ≥5%, ≥10%, and ≥20%. Additional methods for standardizing immunoreactivity analysis included the use of CPS (21%) and TPS (14%).

CPS is defined as the number of positive tumor cells, lymphocytes, and macrophages divided by the total number of viable tumor cells, multiplied by 100.^26^ CPS was employed in three studies,^11,21,22^ with positivity defined as ≥1^11,22^ and ≥20.^21^ TPS corresponds to the number of positive tumor cells divided by the total number of viable tumor cells, multiplied by 100%.^26^ TPS was described in two studies, with positivity determined as ≥1%^18^ and the percentage of neoplastic cells expressing PD-L1 at any intensity. In the latter case, a reference value was not specified.^11^

Table 4 displays the prevalence and association between PD-L1 and HPV status. Among the articles included in this review, PD-L1 immunoreactivity tested positive in 64.28% of the total sample.^11,14,15,17^ Notably, certain studies assessed more than one clone, and both SP142 and 22C3 clones were employed in some instances.^13,14^ In one study, the use of the 22C3 clone was associated with a higher percentage of PD-L1+ cases (64%), while SP142 was correlated with a greater proportion of PD-L1- cases (77%).^14^ Another study reported both SP142 (81.65%) and 22C3 (89.9%) clones as predominantly linked to PD-L1- cases.^13^ Some studies described the immunostaining pattern in both tumor and immune cells,^15,16^ with a higher percentage of PD-L1+ cases observed when immune cells were evaluated (72.82%) compared to tumor cells (53.26%).^15^ In another study, PD-L1+ cases were more common when both immune cells (61.53%) and tumor cells (76.92%) were assessed.^16^

**Table 4:** Prevalence and association between PD-L1 and HPV status.

Study	n	PD-L1 +	PD-L1 -	HPV +	HPV -	HPV +		HPV -		
						PD-L1 +	PD-L1 -	PD-L1 +	PD-L1 -	p-value
Kim et al. (2016)^17^	133	90	43	89	44	63	26	27	17	0.274
Hong et al. (2016)^23^	99	69	30	48	51	40	8	29	22	0.008
Meulenaere et al. (2017)^14^	100	22 (SP142)	77 (SP142)	19	80	11 (SP142)	6 (SP142)	11 (SP142)	66 (SP142)	0.0001 (SP142)
		64 (22C3)	33 (22C3)			10 (22C3)	9 (22C3)	23 (22C3)	55 (22C3)	0.0591 (22C3)
Fukushima et al. (2018)^15^	92	43 (TC)	49 (TC)	45	47	26 (TC)	19 (TC)	17 (TC)	30 (TC)	0.028 (TC)
		67 (IC)	25 (IC)			38 (IC)	7 (IC)	29 (IC)	18 (IC)	0.061 (IC)
Kwon et al. (2018)^24^	79	23	56	28	51	12	16	11	40	0.046
Hong et al. (2019)^20^	214	145	69	81	133	69	12	76	57	0.005
Jeong et al. (2020)^13^	109	17 (SP142)	89 (SP142)	45	61	22 (SP142)	22 (SP142)	22 (SP142)	22 (SP142)	0.043 (SP142)
		8 (22C3)	98 (22C3)			64 (22C3)	64 (22C3)	64 (22C3)	64 (22C3)	0.053 (22C3)
Lilja-Fischer et al. (2020)^11^	303	231	72	156	147	128	28	103	44	0.01
Gurin et al. (2020)^16^	65	50 (TC)	15 (TC)	45	39	24 (TC)	2 (TC)	26 (TC)	13 (TC)	0.0183 (TC)
		40 (IC)	25 (IC)			23 (IC)	22 (IC)	17 (IC)	3 (IC)	0.003 (IC)
Atipas et al. (2023)^22^	160	46	114	27	131	9	18	37	94	0.596
Schmidl et al. (2023)^18^	47	25	16	25	22	12	13	3	19	0.0146
Zhu et al. (2023)^19^	100	56	44	50	50	35	15	21	29	0.005
Lee et al. (2023)^21^	116	45	71	25	91	14	11	31	60	0.046
Kareer et al. (2023)^25^	13	10	3	8	5	8	0	2	3	0.035

IC: Immune Cells, TC: Tumor Cells. The meta-analysis will consider the tumor cell (TC) marking pattern and the SP142 clone (SP142).

Among the articles included in this review, 57.14% were HPV-,^13^ and 78.57% reported more cases of association between PD-L1+ and HPV+ as opposed to PD-L1+ and HPV-.^11,14^ This trend was observed in studies that utilized multiple immunostaining references (TCM and ICM) or clones (SP142 and 22C3).^14^ Only one study employing both the SP142 and 22C3 clones showed an equal number of PD-L1+ and HPV+ cases compared to PD-L1+ and HPV- cases.^13^

### Risk of bias

To assess the risk of bias in the included studies, the JBI Critical Appraisal Checklist for Analytical Cohort Studies was employed. ^12^ All studies received a “yes” for items 1 and 11. However, questions 4 and 5 were designated as “unclear.” Additionally, questions 2, 6, 8, 9, 10, and 11 were considered “not applicable.” For item 3, it was preferable to use multiple methods to determine the presence of HPV and to employ FFPE for IHC evaluation of PD-L1, and most studies received a “no.” In Section 7, the use of CPS or TPS was the standard, but most studies answered “no.” Table 5 presents the assessment for each domain.

**Table 5:** Risk of bias in the studies using the JBI Critical Appraisal Checklist for Analytical Cohort Studies.

Author and year of publication	1	2	3	4	5	6	7	8	9	10	11
Kim et al, 2016^17^	YES	NA	NO	U	U	NA	NO	NA	NA	NA	YES
Hong et al, 2016^23^	YES	NA	NO	U	U	NA	NO	NA	NA	NA	YES
Meulenaere et al, 2017^14^	YES	NA	U	U	U	NA	NO	NA	NA	NA	YES
Fukushima et al, 2018^15^	YES	NA	NO	U	U	NA	NO	NA	NA	NA	YES
Kwon et al, 2018^24^	YES	NA	NO	U	U	NA	NO	NA	NA	NA	YES
Hong et al, 2019^20^	YES	NA	YES	U	U	NA	NO	NA	NA	NA	YES
Jeong et al, 2020^13^	YES	NA	YES	U	U	NA	NO	NA	NA	NA	YES
Lilja-Fischer et al, 2020^11^	YES	NA	NO	U	U	NA	YES	NA	NA	NA	YES
Gurin et al, 2020^16^	YES	NA	U	U	U	NA	NO	NA	NA	NA	YES
Atipas et al, 2023^22^	YES	NA	NO	U	U	NA	YES	NA	NA	NA	YES
Schmidl et al, 2023^18^	YES	NA	NO	U	U	NA	YES	NA	NA	NA	YES
Zhu et al, 2023^19^	YES	NA	NO	U	U	NA	NO	NA	NA	NA	YES
Lee et al, 2023^21^	YES	NA	NO	U	U	NA	YES	NA	NA	NA	YES
Kareer et al, 2023^25^	YES	NA	NO	U	U	NA	NO	NA	NA	NA	YES

NA: not applicable, U: unclear

1 Were the two groups similar and recruited from the same population?

2 Were the exposures measured similarly to assign people to both exposed and unexposed groups?

3 Was the exposure measured in a valid and reliable way?

4 Were confounding factors identified?

5 Were strategies to deal with confounding factors stated?

6 Were the groups/participants free of the outcome at the start of the study (or at the moment of exposure)?

7 Were the outcomes measured in a valid and reliable way?

8 Was the follow-up time reported and sufficient to be long enough for outcomes to occur?

9 Was follow-up complete, and if not, were the reasons to loss to follow-up described and explored?

10 Were strategies to address incomplete follow-up utilized?

11 Was appropriate statistical analysis used?

### Meta-analysis

**Figure 2:**
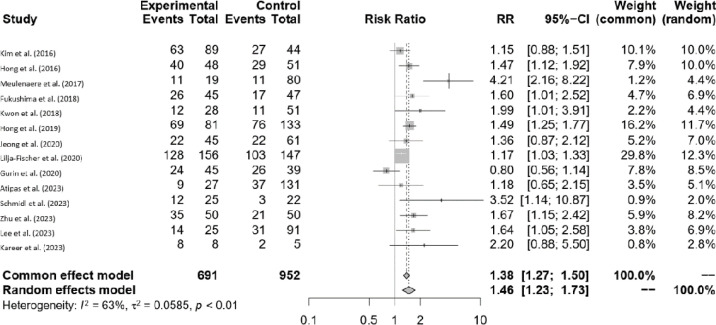
Forest plot related to the included studies.

Statistical analysis was performed using RStudio and Excel 365 software, with calculations carried out using the meta package employing the Mantel-Haenszel method. The difference between the experimental and control groups was statistically significant (p < 0.01), favoring the experimental group, regardless of whether the fixed or random effects model was applied. In terms of heterogeneity, an I2 value of 63% and a p-value of <0 .001 were observed, indicating that the data exhibited heterogeneity. The overall prevalence ratio was 1.38 (1.27; 1.50) for fixed effects and 1.46 (1.23; 1.73) for the random effects model. Notably, two studies^14,18^ reported the highest prevalence ratios (Figure 2).

## Discussion

The hypothesis proposed in this study, suggesting an association between HPV status and PD-L1 immunoexpression in OPSCC, was corroborated by most of the included studies. Furthermore, this hypothesis was confirmed through a meta-analysis.

The primary etiologic factors for OPSCC include smoking, alcohol, and HPV.^4^ Among these, HPV is the dominant etiologic factor,^3^ particularly HPV type 16.^4^ HPV type 16 is responsible for more than 90% of OPSCC cases,^4^ showing significant clinical relevance as HPV+ OPSCC cases generally exhibit a more favorable prognosis compared to HPV- cases.^4,27^ Nevertheless, the exact reasons for this prognostic difference remain uncertain. Some researchers attribute the better prognosis of HPV+OPSCC to heightened sensitivity to chemoradiotherapy.^28^

Furthermore, the expression of tumor suppressor gene p16 has been found to correlate with HPV infection in OPSCC.^29^ It is believed that p16 overexpression results from the viral E7 components, which interfere with Rb function, ultimately upregulating p16. Therefore, IHC staining of p16 is commonly used as a surrogate marker for HPV status in OPSCC.^30,31^

HPV-induced tumorigenesis is closely linked to chronic inflammation caused by the viral process. Immune responses triggered by HPV infection differ from those associated with tobacco-induced carcinogenesis. The immune system plays a main role in eradicating tumors, but cancer cells employ various mechanisms to evade detection or disrupt the immune system. These mechanisms include the development of T-cell tolerance, modification of HLA class I, inhibition of inflammatory cytokines, and evasion of immune checkpoint processes.^32^ As a result, components of the tumor microenvironment, including lymphocytes, macrophages, and immune checkpoints, are critical in either inhibiting or promoting cancer cell development.^33,34^

PD-1 is a transmembrane receptor expressed by T cells, B cells, monocytes, and dendritic cells, and it plays a role in the immune checkpoint cascade. The binding of PD-1 to PD-L1 on tumor cells helps these cells evade immune surveillance.^32^ The expression of PD-L1 on both cancer cells and immune cells is associated with survival outcomes and responses to immune checkpoint inhibitors.^17,35^ The regulation of PD-L1 in oropharyngeal tumor cells is considered an adaptive immune response in chronic diseases associated with viral infection.^14^ In some studies, high immunoexpression of PD-L1 is observed in HPV-associated cancers.^35^

Understanding the TME in OPSCC is crucial because the presence of HPV is correlated with a more favorable prognosis,^4,27^ and HPV is the primary etiologic factor for this type of tumor.^4^ Furthermore, PD-1 and PD-L1 are immunoregulatory factors.^8^ In OPSCC, high intratumoral expression of PD-L1 in immune cells can identify subgroups of patients with an excellent response to cancer treatment and offer prognostic value.^11^ The immunoexpression of PD-L1 in the TME in HPV OPSCC can help identify patients eligible for immunotherapy,^9,10^ providing a therapeutic approach with fewer adverse effects and improved survival.^36^ Note that the response to immunotherapy differs between patients with HPV+ and HPV-OPSCC,^6,7^ in line with the finding of the present research, which links HPV+ to positive PD-L1 immunoexpression in OPSCC.

Epidemiologically, this review concludes that adult men are more commonly affected by OPSCC, concurring with the literature, which describes a male-to-female ratio of 6:1^37^ and diagnosis at an age younger than 60 years.^38^ The tonsils were the most common site in those studies that stratified anatomical location.^11,13^ Tonsillar squamous cell carcinoma (TSCC) accounts for 70%-80% of all OPSCC cases. Note that TSCC is an aggressive tumor with early lymphatic spread and a poor prognosis.^24^ Risk factors for OPSCC include genetic, environmental, and behavioral factors, with an emphasis on tobacco (smoked and smokeless), excessive alcohol consumption, and a potential association with HPV.^39^ In this review, most of the sample consisted of smokers and alcohol consumers.

Staging may vary depending on the reference used, such as TNM and AJCC, and it is recommended that both be analyzed. The eighth edition of the AJCC staging system, published in 2017, is the most recent one. However, the seventh edition is still commonly used, and the main difference between the editions lies in the introduction of depth of invasion (DOI). DOI reflects the proximity of the tumor to the underlying lymphovascular tissues and has been associated with nodal metastasis.^30^ Among the selected articles, the seventh edition of AJCC was more frequently utilized, even in studies conducted after 2017. The studies consistently demonstrated a predominance of advanced staging, irrespective of the assessment method employed. Additionally, it was observed that the N category was generally ≥2, which may be attributed to the high number of TSCC cases, as these tumors often exhibit early lymphatic spread.

Several histologic subtypes of OPSCC exist, including HPV-associated non-keratinizing squamous cell carcinoma; HPV-associated keratinizing squamous cell carcinoma; HPV-associated papillary squamous cell carcinoma; HPV-associated adenosquamous carcinoma; HPV-associated ciliated adenosquamous carcinoma; HPV-associated lymphoepithelial carcinoma; HPV-associated spindle cell/sarcomatoid squamous cell carcinoma; and HPV-associated basoid squamous cell carcinoma.^40^

In 2017, the World Health Organization (WHO) established that HPV+ OPSCC exhibits a non-keratinizing morphology distinct from the morphology of squamous cell carcinomas (SCC) associated with alcohol and tobacco . These associations of factors may help to explain the tendency for patients affected by this HPV-related malignancy to often have a better prognosis and longer survival (90%) than traditional keratinizing SCC.^41^ In the Bluebook published by WHO in 2022, the histologic progression from sequential stages of surface dysplasia to carcinoma in situ and invasive growth, typically observed in HPV-independent SCC, is not evident for HPV-associated OPSCC.^40^

Instead, tumors originate from the tonsillar crypts and infiltrate beneath the surface epithelium, forming nests and lobules, often with central necrosis. Invasive growth may not induce a desmoplastic stromal reaction, and because the reticulated tonsillar crypt epithelium is a poor barrier to spread, SCC adjacent to organized lymphoid tissue can metastasize, despite appearing histologically only in situ. The tumor nests tend to be surrounded by a lymphoid stroma that may permeate the tumor lobules. In the typical tumor, tumor cells display high N:C ratios, oval to spindled nuclei, and syncytial cytoplasm (indistinct cell borders) without intercellular bridges, often lacking cytoplasmic keratinization. These cellular features are termed “non-keratinizing SCC.” Histologic grading has been shown to lack prognostic utility and is not advocated. Some tumors may show nuclear anaplasia or multinucleation. However, the Bluebook published in 2022 describes non-keratinizing SCC morphology as one of the essential and desirable diagnostic criteria.^40^ Unfortunately, the studies included in this review did not mention this classification, referring to tumor grades as well, moderate, poor, or basaloid or not providing any information whatsoever.

More than 90% of OPSCC cases are associated with high-risk HPV type 16.^4^ In this systematic review, all studies specifying the HPV type mentioned type 16. However, most of the sample was HPV- (57.14%), which could be linked to the method used for HPV detection. HPV can be identified through various methods, including detection of viral mRNA, detection of viral DNA with PCR, detection of viral DNA without PCR, detection of viral DNA with ISH, detection of indirect markers of HPV-induced carcinogenesis (e.g., p16 protein, pRb, p53, cyclin D1), and detection of antibodies against HPV antigens in serum. The diagnostic gold standard for HPV-related OPSCC, focusing on E6/E7 mRNA detection, requires fresh samples. Because most frequently available samples are FFPE, IHC is often indicated.^30^

A recent literature review on HPV identification methods showed greater sensitivity in RNA ISH, DNA PCR, p16 IHC, and DNA ISH, in descending order. Regarding specificity, DNA ISH, p16 IHC, RNA ISH, and DNA PCR were mentioned. Although PCR and ISH are strongly indicated for identifying HPV, p16 IHQ correlates well with HPV DNA and can serve as a surrogate marker for HPV.^42^ P16 IHQ status has also been adopted by the AJCC 8th edition as a proxy for HPV-relatedness in OPSCC staging. However, p16 IHQ still lacks specificity for transcriptionally active HPV even though it has been shown to be a good surrogate marker for HPV positivity.^21^ Concurrently, the WHO Bluebook published in 2022 outlines the positivity for high-risk HPV as essential and desirable diagnostic criteria for HPV-associated OSCC, based on IHCp16 (70% of the nuclear and cytoplasmic cutoff) or IHCp16, coupled with specific HPV tests .^40^ Therefore, it is advisable to apply more than one assessment method rather than relying solely on IHC.^21,30,40,42^

The analytic methods mentioned in the articles included IHC (50%), real-time PCR (28.57%), ISH (14.28%), PANArray HPV chip (7.14%), a combination of IHC with real-time PCR (7.14%), and a combination of IHC with ISH (7.14%). In this research, five articles (42.85%) had a higher prevalence of HPV+ in their samples when HPV detection was performed exclusively by IHC,^11,17,18,25^ and in one study, the method was not reported.^16^ One study used IHC and PCR, and obtained more HPV- cases.^20^ The study that applied IHC and ISH showed more than half of the cases were HPV+,^18^ but in the study that exclusively used ISH, less than a fifth of the sample was HPV+.^14^ These results underscore the importance of utilizing at least two evaluation methods.

As far as the immunohistochemical expression of PD-L1 is concerned, a systematic review comparing commercially available PD-L1 clones concluded that SP142, SP263, and E1L3N are the most reliable ones and thatE1L3N is the preferred clone among the three.^43^ In the articles included in our review, the clones used included SP142, 22C3, 31L3N, 5H1, 28-8, SP263, and CAL10, and E1L3N was used in only two studies. Both studies using E1L3N had significantly higher PD-L1 immunoexpression compared to PD-L1-.^20^

Only two studies used TMA as the processing technique. While this approach streamlines laboratory work, it can introduce variability in PD-L1 immunoexpression. This is because the antibody exhibits a heterogeneous staining pattern within the tumor, and selecting a small area may lead to inaccurate conclusions.^44^ In this review, the two authors who used TMA had a higher amount of PD-L1- in their samples, even when employing SP142^24^ and SP263.^22^

To assessing PD-L1 immunoexpression via CPS and TPS is a suitable option for determining anti-PD-1/PD-L1 therapy. CPS is superior because the predictive value of PD-L1 expression increases when its combined expression is considered in both TC and IC (≥ 1). PD-L1 labeling in TC is characterized by a close relationship between TC and IC within the TME.^45^ Among the selected studies, only three used CPS, with values of ≥1^11,22^ and ≥ 20.^21^ Furthermore, most of the studies^13^ relied on visual inspection with different parameters, not allowing for a standardized analysis.

The limitations of this systematic review include the fact that the studies were conducted in countries with similar cultural backgrounds, used less robust study designs, and did not distinguish between keratinizing and non-keratinizing histologic morphology. Few studies identified HPV by in situ hybridization, the PD-L1 clones used exhibited heterogeneity, and dilution and immunoexpression patterns varied considerably. Additionally, some studies had small sample sizes. A notable limitation was the high risk of bias in most studies, with many responses rated as “no” for the items “Was the exposure measured validly and reliably?” and “Were the results measured validly and reliably?”. Further research is required to provide additional evidence regarding the association between HPV status and PD-L1 immunoexpression in OPSCC.

## Conclusion

In conclusion, HPV positivity is linked to positive PD-L1 immunoexpression in OPSCC. Therefore, patients diagnosed with HPV+ OPSCC tend to have a higher PD-L1 expression. In this way, it is possible to implement immunotherapy treatment targeted at this protein. Such therapy has a greater chance of clinical success and fewer harmful effects than other oncologic interventions.
